# Cognitive Profiles and Functional Connectivity in First-Episode Schizophrenia Spectrum Disorders – Linking Behavioral and Neuronal Data

**DOI:** 10.3389/fpsyg.2019.00689

**Published:** 2019-04-02

**Authors:** Mabel Rodriguez, Yuliya Zaytseva, Aneta Cvrčková, Boris Dvořaček, Aneta Dorazilová, Juraj Jonáš, Petra Šustová, Veronika Voráčková, Marie Hájková, Zuzana Kratochvílová, Filip Španiel, Pavel Mohr

**Affiliations:** ^1^National Institute of Mental Health, Klecany, Czechia; ^2^Department of Psychology, Faculty of Arts, Charles University in Prague, Prague, Czechia; ^3^Third Faculty of Medicine, Charles University in Prague, Prague, Czechia; ^4^Department of Psychology, Faculty of Social Studies, Masaryk University, Brno, Czechia; ^5^Department of Psychology, Faculty of Arts, Masaryk University, Brno, Czechia

**Keywords:** cognitive deficit, schizophrenia, first episodes, cognitive profiles, heterogeneity, cluster analysis, resting state functional connectivity

## Abstract

The character of cognitive deficit in schizophrenia is not clear due to the heterogeneity in research results. In heterogeneous conditions, the cluster solution allows the classification of individuals based on profiles. Our aim was to examine the cognitive profiles of first-episode schizophrenia spectrum disorder (FES) subjects based on cluster analysis, and to correlate these profiles with clinical variables and resting state brain connectivity, as measured with magnetic resonance imaging. A total of 67 FES subjects were assessed with a neuropsychological test battery and on clinical variables. The results of the cognitive domains were cluster analyzed. In addition, functional connectivity was calculated using ROI-to-ROI analysis with four groups: Three groups were defined based on the cluster analysis of cognitive performance and a control group with a normal cognitive performance. The connectivity was compared between the patient clusters and controls. We found different cognitive profiles based on three clusters: Cluster 1: decline in the attention, working memory/flexibility, and verbal memory domains. Cluster 2: decline in the verbal memory domain and above average performance in the attention domain. Cluster 3: generalized and severe deficit in all of the cognitive domains. FES diagnoses were distributed among all of the clusters. Cluster comparisons in neural connectivity also showed differences between the groups. Cluster 1 showed both hyperconnectivity between the cerebellum and precentral gyrus, the salience network (SN) (insula cortex), and fronto-parietal network (FPN) as well as between the PreCG and SN (insula cortex) and hypoconnectivity between the default mode network (DMN) and seeds of SN [insula and supramarginal gyrus (SMG)]; Cluster 2 showed hyperconnectivity between the DMN and cerebellum, SN (insula) and precentral gyrus, and FPN and IFG; Cluster 3 showed hypoconnectivity between the DMN and SN (insula) and SN (SMG) and pallidum. The cluster solution confirms the prevalence of a cognitive decline with different patterns of cognitive performance, and different levels of severity in FES. Moreover, separate behavioral cognitive subsets can be linked to patterns of brain functional connectivity.

## Introduction

Schizophrenia is typically described as a heterogeneous disease (Tandon et al., 2009; Owen et al., 2016). A model of heterogeneity in Schizophrenia with its causes, characteristics, and course and outcome was well summarized by Seaton et al. (2001). Symptoms of schizophrenia, treatment response, and outcome vary during the course of the illness (Andreasen et al., 2005; Tandon et al., 2009). The heterogeneity has also its implication for the better understanding of its pathogenesis (Liang and Greenwood, 2015); it complicates also the search for neurobiological correlates and the character of cognitive functioning (Heilbronner et al., 2016; Owen et al., 2016).

Cognitive deficit (CD) is a well-established marker of schizophrenia (Heinrichs and Zaksanis, 1998; Mesholam-Gately et al., 2009; Fioravanti et al., 2012), observed already in the early stage of the illness (Fusar-Poli et al., 2012), and before the patient is exposed to medication (Fatouros-Bergman et al., 2014). Moreover, CD is present in ultra-high risk states (Bora and Murray, 2014), and other psychotic disorders (Bora et al., 2009, 2010) and influences daily functioning (Bowie et al., 2006). Results of research show a great variability of cognitive functioning in schizophrenia: (1) CD over time can be either stable, declining, or improving (Szöke et al., 2008); (2) CD is general or partial (Heilbronner et al., 2016); (3) different cognitive phenotypes can be considered as endophenotypes or predictors of daily functioning (Szöke et al., 2008; Wölwer et al., 2008). Factorial analyses of cognition in schizophrenia consistently show a deficit in six-to-seven cognitive factors (domains): attention, verbal, visual, and working memory (WM), speed of processing (APOP), reasoning and abstraction (ABST), and social cognition (Nuechterlein et al., 2004, 2008; Dickinson et al., 2006; Rodriguez et al., 2017). Moreover, neuroimaging studies have found a correlation between structural and functional brain analysis and cognition. One of the most important theories in this relationship is the dysconnectivity theory of schizophrenia, which implies an abnormal pattern of connections among distinct brain regions, referred as cognitive dysmetria. This disrupted connectivity results in altered functional integration since it involves either exaggerated connections or weakened pathways (Andreasen et al., 1996; Stephan et al., 2006; Fornito et al., 2011). A more recent systematic review of cognition and resting-state functional connectivity in schizophrenia found abnormalities within and between regions such as the cortico–cerebellar–striatal–thalamic loop and task-positive and task-negative cortical networks. But they did not observe unique relationships between specific functional connectivity abnormalities and distinct cognitive domains. The authors acknowledge as one of the limitations, the inter-dependency of many neuropsychological tasks on multiple cognitive processes, making it difficult to identify what cognitive functions are truly impaired in schizophrenia and the non-specific patterns of resting-state functional connectivity correlations with distinct cognitive domains (Sheffield and Barch, 2016). Therefore, while there is no doubt about the presence and impact of CD in schizophrenia, the character of the deficit remains unclear. New approaches are necessary to help us better understand the nature of cognitive functioning in schizophrenia.

In order to study the character of a heterogeneous disorder, it is useful to establish more homogenous groups inside the condition. Cluster analysis is a method that allows the classification of individuals based on their profiles, helping to reduce the heterogeneity of findings. In psychotic disorders, a cross-diagnostic cluster analysis in subjects with schizophrenia, schizoaffective disorder, and bipolar disorder with psychosis provided four different cognitive profiles. The diagnoses were distributed among all cognitive clusters and the authors concluded that specific neurocognitive profiles can underlie relevant neural abnormalities between these diagnoses (Lewandowski et al., 2014). In schizophrenia, the identification of distinct phenotypes of cognitive profiles better explains the relationship between cognitive profiles and external variables (Seaton et al., 2001) such as age, education (Goldstein et al., 1998), clinical symptomatology (Hill et al., 2002), or functional outcome (Gilbert et al., 2014). Another approach to reducing the heterogeneity of results with clusters is to examine the relationships within cognitive variables and neuroimaging procedures, for example between brain function and structure (Heinrichs and Awad, 1993; Geisler et al., 2015), or as a phenotypic feature shared with first-degree relatives (Ohi et al., 2017).

Cluster analysis of cognitive functioning in schizophrenia typically generates three clusters: mild (like-normal), moderate (partial deficit), and severe (general deficit). The number of cognitive clusters has been demonstrated to be more or less consistent, but the heterogeneity persists within the clusters. A more homogeneous sample and a combination of different external variables may help to understand the nature of the profiles.

The aim of our study was to examine cognitive profiles of first-episode schizophrenia spectrum disorders (FES) based on cluster analysis of their cognitive performance, and to correlate these profiles with clinical variables and resting state brain connectivity, as measured with magnetic resonance imaging.

## Materials and Methods

The study was conducted at the National Institute of Mental Health, Czech Republic; the study protocol was approved by the Ethical Committee.

### Study Population

The study subjects were diagnosed with FES according to ICD-10 criteria with F20 (55%) and F23 (45%). Patients were evaluated under a clinically stable condition, at the end of their first psychiatric hospitalization, and in a partial symptomatic remission state, according to Andreasen’s remission criteria (2005). The study subjects were diagnosed in a routine clinical process by two experienced psychiatrists. In the event of diagnostic disagreements (e.g., comorbidity), the subjects were excluded from the study. Other exclusion criteria were: neurological disorders, head injuries, comorbid psychiatric or somatic disorder, specific developmental disorders of academic skills, and other medical or cognitive conditions that may alter cognitive functioning, metal implants in the head and face, or a cardiac pacemaker. The control group consisted of healthy subjects (HC), without history of any psychiatric disorders. All of the study subjects signed an informed consent form.

### Study Materials and Procedures

#### Neuropsychological Assessment

**FIGURE 1:**
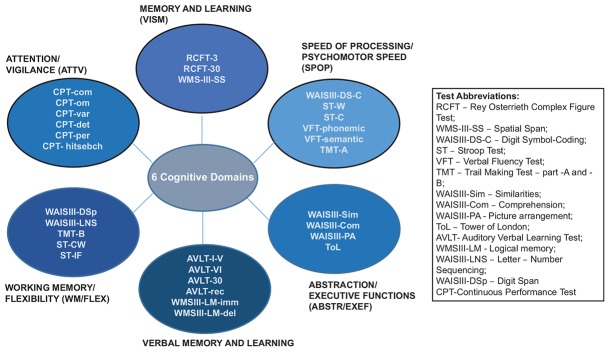
Composition of the cognitive domains (Rodriguez et al., 2017).

The study subjects were administered a battery of neuropsychological tests, consisting of the following domains (for a detailed description of the calculation of the composition and consistency of cognitive domains, see Rodriguez et al., 2017): SPOP, attention/vigilance (ATTV), WM/flexibility (FLEX), VERBM, visual memory (VISM), and ABST/executive functions (/EXEF). The composition of cognitive domains is presented in Figure 1. The mean duration of the neuropsychological assessment was 150 min. The participants were tested in two consecutive sessions with a break in between. The cognitive assessment was conducted within a maximum of 2 years after the first psychotic episode and within 1 week of imaging assessment. The cut-off on the cognitive domains for our study was *SD*±1.0.

#### Demographic Variables of the Sample

The assessment of FES included the positive and negative syndrome scale (PANSS; Kay et al., 1987), and the short-form quality of life (QOL) questionnaire WHOQOL-BREF (The WHOQOL Group, 1998), the Czech version validated for the Czech population (Dragomirecká and Bartonová, 2006). Demographic data [age, education, gender, employment status, duration of untreated psychosis (DUP), and medication dosage in chlorpromazine equivalents (CHLPZ)] were obtained during the clinical structured interview by a trained psychiatrist.

For the subsequent analysis of the corresponding brain functional connectivity patterns in three cluster groups, we included a control group that consisted of 20 healthy individuals selected randomly from the overall control group used in behavioral analysis (*z*-scores).

#### Neuroimaging Data Acquisition and Preprocessing

The magnetic resonance images were acquired at two sites: National Mental Health Institute Klecany (NUDZ Klecany, site 1) and Institute of Clinical and Experimental Medicine Prague (IKEM Prague, site 2). The percentage of the subjects scanned at each site was 41% and 59%, respectively.

The structural and functional scans from site 1 were acquired on a Siemens MAGNETOM Prisma 3T. The T1-weighted images (T1W) with the optimized magnetization prepared by Rapid Acquisition Gradient Echo (MPRAGE) had 224 scans with a slice thickness of 0.7 mm, repetition time 2,400 ms, echo time 2,34 ms, inversion time 1,000 ms, flip angle 8°, and acquisition matrix 320 × 320 mm. The T2^∗^-weighted images contained 300 scans with a slice thickness of 3 mm, repetition time 2,000 ms, echo time 30 ms, acquisition matrix 64 × 64, and flip angle 70°.

The images from IKEM Prague were acquired on a Siemens Trio Tim 3T: T1W images with repetition time 2,300 ms, echo time 4.63 ms, inversion time 900 ms, slice thickness 1 mm, flip angle 10°, and acquisition matrix 256 × 256 mm; T2^∗^-weighted images – 300 scans with slice thickness 3 mm, repetition time 2,000 ms, echo time 30 ms, acquisition matrix 64 × 48, and flip angle 70°.

Resting state scans were acquired with eyes closed. All of the subjects were instructed to relax and keep their head still. To ensure that the subjects were comfortable and to minimize the head motion, each of them was provided with a head support and padding.

Individual resting state scans were pre-processed with SPM 8 and CONN v.17 Toolbox (Whitfield-Gabrieli and Nieto-Castanon, 2012). The data underwent standard pre-processing. The functional images were slice-timed, spatially normalized, co-registered to the respective structural scans, smoothed using a Gaussian kernel of FWHM of 8 mm, and band pass filtered (0.008–0.09 Hz). Further, for the connectivity analysis, the data underwent further steps of preprocessing in CONN: denoizing [the original distribution of the connectivity values before denoizing was at a maximum (0.48±0.23, *df* = 399.0)], after denoizing (0.02±0.16, *df* = 122.7), and CompCor algorithm discarding motion-related signal changes coming from voxels within white matter and cerebrospinal fluid in the ventricles, cardiac and respiratory fluctuations, and effects of head motion (for details, see Behzadi et al., 2007).

### Statistical Analysis

#### Cluster Solution

For cluster calculation, the raw performance scores in the cognitive tests were converted into *z*-scores. The *z*-scores were calculated as the difference among the raw scores of the study subject and the sample mean of a larger sample of healthy controls (*N* = 90), divided by the standard deviation of the healthy sample. The cumulative test score of each cognitive domain was calculated as a mean of *z*-scores. The hierarchical cluster analysis (Ward’s cluster method with squared Euclidean distance measurements) was applied as a classification procedure in order to identify groups of study subjects with similar cognitive profiles.

#### Demographic Variables of the Sample

Demographic variables: gender, education, and time of cognitive assessment were tested using Fischer’s exact test. Age variable, clinical variables DUP, symptomatology, CHLPZ, and QOL were tested with the Kruskal–Wallis test.

#### Statistical Analysis of Resting State Functional Neuroimaging Data

Functional connectivity was computed using ROI-to-ROI analysis. The ROIs or NOIs were defined by means of the functional seeds and networks (158 ROIs) derived from standardized atlases: atlas.nii/.txt/.info, an atlas of cortical and subcortical areas from the FSL Harvard–Oxford Atlas, as well as cerebellar areas from the AAL atlas and (2) networks.nii/.txt/.info, an atlas of a few commonly used networks and areas (e.g., Default Mode MPFC/PCC/RLP/LLP areas).

In order to evaluate the average effect, a statistical analysis implied MANCOVA with four groups was performed: three groups were defined based on the behavioral cluster analysis and cognitive performance (three groups with respect to three defined clusters) and a control group of healthy individuals with a normal cognitive performance (the controls) in order to evaluate the average effect. A scanner type (1-NUDZ, 0_IKEM) was applied as a covariate. Furthermore, a *post hoc* analysis was performed to test between-group differences only in the brain seeds that were found to be significant in the original *F*-test. Multiple comparisons were thresholded at *p* < 0.05 FDR. Results were presented with an uncorrected *p* < 0.001 when no results were obtained with an FDR-corrected *p* < 0.05.

## Results

A total of 67 FES subjects were enrolled in this study. Their ages ranged from 17 to 54 (mean, *M* = 29.4, *SD* = 7.5). The sample was composed of *n* = 36 men (53.7%) and *n* = 31 women (46.3%). The level of education was: *n* = 10 (14.9%) elementary school; *n* = 20 (29.9%) vocational school; *n* = 16 (23.9%) high school; *n* = 21 (31.3%) university. The percentage of subjects diagnosed with F20 was 55% (*N* = 37) and with F23 was 45% (*N* = 30). There was no difference between the subjects diagnosed with F20 and F23.1 in terms of their demographics (Table 1).

**Table 1:** Descriptive characteristics of the sample according to diagnosis (F20 vs. F23).

		F20 *N* = 37	F23 *N* = 30	χ^2^ (df)	Cramer’s *V*
Gender	Male	22	14	0.64 (1), *p* = 0.43	0.097
	Female	15	16		
Education	Elementary	5	5	1.58 (3), *p* = 0.66	0.15
	Vocational	10	10	
	High	11	5		
	University	11	10		
				*t* (df)	Cohen’s *d*

Age	Mean	28.76	30.13	-0.73(55.75), *p* = 0.47	-0.18
	(*SD*)	(6.76)	(8.27)		
	Range	17–45	18–54		

The majority of the sample was assessed within the first year after the onset of the illness (*n* = 48, 71.6%). The cognitive performance in the FES group did not significantly differ between the subjects evaluated during the first year after the onset and those evaluated during 2 years after the first psychotic episode {VISM [*F*(1.65) = 1.06, *p* > 0.05]; VERBM [*F*(1.65) = 0.01, *p* > 0.05]; SPOP [*F*(1.65) = 0.50, *p* > 0.05]; ABSTR [*F*(1.65) = 0.35, *p* > 0.05]; FLEX [*F*(1.65) = 0.00, *p* > 0.05]; ATTV [*F*(1.65) = 0.13, *p* > 0.05]}.

### Cluster Solution

**FIGURE 2:**
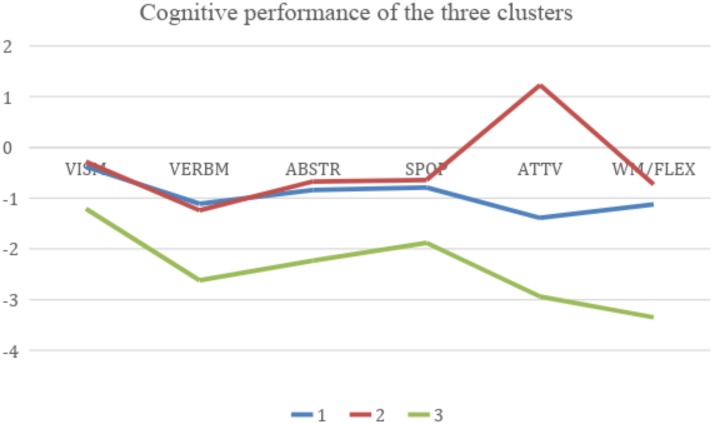
Cognitive performance of the three clusters. Blue line Cluster 1: decline in the VERBM and WM/FLEX domains (*z*-scores below point -1.0). Red line Cluster 2: decline in the VERBM domain only (*z*-scores below point -1.0) and above average performance in the ATTV domain (*z*-scores over point 1.0). Green line: Cluster 3: generalized and severe impairment in all cognitive domains (*z*-scores ranging from -1.21 in the VISM domain to -3.35 in the WM/FLEX domain). *Domains:* VISM, visual memory and learning; VERBM, verbal memory and learning; ABSTR/EXEF, abstraction/executive functions; SPOP, speed of processing/psychomotoric speed; ATTV, attention/vigilance; WM/FLEX, working memory/flexibility.

The hierarchical cluster analysis (Ward’s cluster method with squared Euclidean distance measurements) on the three predefined clusters of our sample confirmed the existence of various cognitive profiles with different performance levels. The first cluster was represented by a group of FES subjects with a decline in the VERBM domain and the WM/FLEX domain (*z*-scores below point -1.0). The second cluster was represented by a group of FES subjects with a decline in the VERBM domain only (*z*-scores below point -1.0) and contrarily this group performed above average in the ATTV domain (*z*-scores over point 1.0). The third cluster presented a generalized and severe impairment in all cognitive domains (*z*-scores ranging from -1.21 in the VISM domain to -3.35 in the WM/FLEX domain). The cognitive performance of these three clusters is presented in Figure 2 and the descriptive characteristics of the cognitive domains of the three clusters are presented in Table 2.

**Table 2:** Descriptive characteristics of the cognitive domains of the three clusters.

Cluster	VISM		VERBM		ABSTR		SPOP		ATTV		FLEX	
	*M*	*SD*	*M*	*SD*	*M*	*SD*	*M*	*SD*	*M*	*SD*	*M*	*SD*
1	-0.38	0.75	-1.11	0.99	-0.84	0.85	-0.79	0.59	-1.39	0.98	-1.12	0.87
2	-0.28	0.83	-1.24	1.06	-0.67	1.05	-0.64	0.69	1.23	0.89	-0.73	0.95
3	-1.21	0.58	-2.62	0.9	-2.23	0.45	-1.88	0.75	-2.94	0.96	-3.35	1.65

The clusters did not differ in terms of their demographic variables (gender, education, time of cognitive assessment tested using Fischer’s exact test, and age variable tested using the Kruskal–Wallis test), clinical variables (DUP, PANSS, CHLPZ), and QOL (WHOQOL-BREF). The results are presented in Table 3. In addition, there was no difference between the subjects diagnosed with F20 and F23.1 in terms of their cognitive median (Table 4) or cluster distribution (Table 5).

**Table 3:** Demographic variables of the samples according to cluster distribution.

		Cluster 1 (*N* = 35)	Cluster 2 (*N* = 24)	Cluster 3 (*N* = 8)		
					***p*-value**	**Cramer’s *V***
	
Gender	Male	15	17	4	0.1	0.26
	Female	20	7	4		
Education	Elementary	5	2	3	0.45	0.22
	Vocational	10	8	2		
	High school	10	4	2		
	University	10	10	1		
Time of cognitive						
assessment	Within 1 year	25	17	6	1	0.03
	Within 2 years	10	7	2		
	
					K–W test	𝜀^2^
	
Age	Mean	29.2	28.83	31.75	*H*(2) = 0.09, *p* > 0.05	0.00
	(*SD*)	(6.15)	(6.69)	(13.58)		
	Range	18–43	25–40	19–54		
DUP	Mean	3.25	2.5	1.88	*H*(2) = 0.98, *p* > 0.05	0.02
	(*SD*)	(4.77)	(4.26)	(2.01)		
	Range	0–21	0–15	0–5		
CHLPZ	Mean	415.73	346.83	424	*H*(2) = 0.58, *p* > 0.05	0.001
	(*SD*)	(298.39)	(209.87)	(203.48)		
	Range	114–1333	7–883	228–755		
PANSS	**Positive**				*H*(2) = 2.46, *p* > 0.05	0.04
	Mean	10.82	12	13.75		
	(*SD*)	(2.54)	(4.08)	(4.92)		
	Range	7–16	7–20	9–23		
	**Negative**				*H*(2) = 0.48, *p* > 0.05	0.001
	Mean	14.95	15.04	15.88		
	(*SD*)	(5.45)	(4.99)	(5.99)		
	Range	7–28	7–29	7–25		
	**General**				*H*(2) = 0.53, *p* > 0.05	0.001
	Mean	28.71	28.92	31.12		
	(*SD*)	(5.8)	(6.17)	(9.11)		
	Range	19–41	18–39	19–44		
	**Total**				*H*(2) = 1.57, *p* > 0.05	0.02
	Mean	54.47	55.96	60.75		
	(*SD*)	(11.39)	(12.14)	(17.16)		
	Range	35–75	37–72	35–83		
WHOQOL-BREF	**Dom1**				*H*(2) = 3.16, *p* > 0.05	0.05
	Mean	14.23	14.98	13.07		
	(*SD*)	(2.92)	(1.75)	(2.53)		
	Range	9.14–19.43	11.43–18.29	9.71–17.14		
	**Dom2**				*H*(2) = 2.60, *p* > 0.05	0.04
	Mean	13.04	14.31	14.66		
	(*SD*)	(3.29)	(2.31)	(1.82)		
	Range	5.33–18	10.67–20	11.33–17.33		
	**Dom3**				*H*(2) = 0.63, *p* > 0.05	0.01
	Mean	13.8	13.17	13.67		
	(*SD*)	(3.38)	(2.4)	(2.55)		
	Range	8–20	9.33–17.33	9.33–17.33		
	**Dom4**				*H*(2) = 2.25, *p* > 0.05	0.03
	Mean	14.81	15.56	14.94		
	(*SD*)	(2.17)	(2.0)	(2.23)		
	Range	10.5–20	11.5–19.5	12.5–18.5		

**Table 4:** Comparison of cognitive performance between subjects diagnosed with F20 and F23.

Domain	F20 group	F23 group		
	*M* (*SD*)	*M* (*SD*)	*W*	*p*
VISM	-0.44 (0.88)	-0.45 (0.71)	559	0.97
VERBM	-1.2 (1.1)	-1.51 (1.09)	652	0.23
ABSTR/EXEF	-0.87 (0.99)	-1.04 (1.04)	615	0.46
SPOP	-0.98 (0.73)	-0.73 (0.75)	470	0.29
ATTV	-0.79 (1.74)	-0.44 (1.79)	486	0.39
WM/FLEX	-1.27 (1.44)	-1.22 (1.07)	609	0.50

**Table 5:** Comparison of cluster distribution between subjects diagnosed with F20 and F23.

		F20	F23	*c*^2^ (df)	Cramer’s *V*
Clusters	1	20	15	0.15 (2), *p* = 0.93	0.048
	2	13	11		
	3	4	4		

#### Resting State Connectivity Results

For the subsequent analysis of the corresponding brain functional connectivity patterns in the three cluster groups, we included a control group that consisted of 20 healthy individuals randomly selected from the larger healthy control sample included in the behavioral analysis (*z*-scores). The groups did not differ in terms of their demographic (Table 6), but education (Table 7) characteristics. There were no differences between groups on cognitive domain characteristics (Table 8). The distribution of the subjects scanned at each site (site 1 and site 2) was as follows: 40% and 60% for Cluster 1; 41% and 59% for Cluster 2; 50% and 50% for Cluster 3; and 50% and 50% for Cluster 4 (control group).

**Table 6:** Demographic comparison between the selected group for brain functional connectivity analysis and the larger healthy control sample.

		MRI	COG	χ^2^ (df)	Cramer’s *V*
Gender	Male	9	32	0 (1), *p* = 1	0.00
	Female	11	37		
				Fisher’s exact test	
Education	Elementary	0	6	*p* = 0.12	0.27
	Vocational	0	3		
	High	8	38		
	University	12	22		
				*t* (df)	Cohen’s *d*

Age	Mean	29.1	29.04	0.03 (30.53), *p* = 0.98	0.01
	(*SD*)	(7.25)	(7.15)		
	Range	22–44	16–45		

*MRI, randomly selected group for brain functional connectivity analysis; COG, larger healthy control sample included in the behavioral analysis (z-scores).*

**Table 7:** Demographic comparison between the selected group of HC for brain functional connectivity analysis and the patients sample.

		MRI	FES	χ^2^ (df)	Cramer’s *V*
Gender	Male	9	36	0.19 (1), *p* = 0.66	0.05
	Female	11	31		
				Fisher’s exact test	
Education	Elementary	0	10	*p* = 0.001	0.39
	Vocational	0	20		
	High	8	16		
	University	12	21		
				*t* (df)	Cohen’s *d*

Age	Mean	29.1	29.37	-0.14 (31.92), *p* = 0.88	-0.03
	(*SD*)	(7.25)	(7.55)		
	Range	22–44	17–54		

*MRI, randomly selected group for brain functional connectivity analysis; FES, patients sample.*

**Table 8:** Cognitive domain comparison between the selected group for brain functional connectivity analysis and the larger healthy control sample.

	MRI group	Cognition group		
	*M* (*SD*)	*M* (*SD*)	*W*	*p*
VISM	-0.17 (0.76)	0.04 (0.79)	573	0.25
VERBM	0.07 (0.69)	-0.02 (0.74)	662	0.67
ABSTR/EXEF	0.1 (0.79)	-0.02 (0.77)	711	0.51
SPOP	0.01 (0.56)	0.01 (0.62)	683	0.78
ATTV	0.2 (0.56)	-0.07 (0.75)	830	0.11
WM/FLEX	0.13 (0.8)	-0.04 (0.62)	731	0.45

*MRI, randomly selected group for brain functional connectivity analysis; COG, larger healthy control sample included in the behavioral analysis (z-scores).*

**FIGURE 3:**
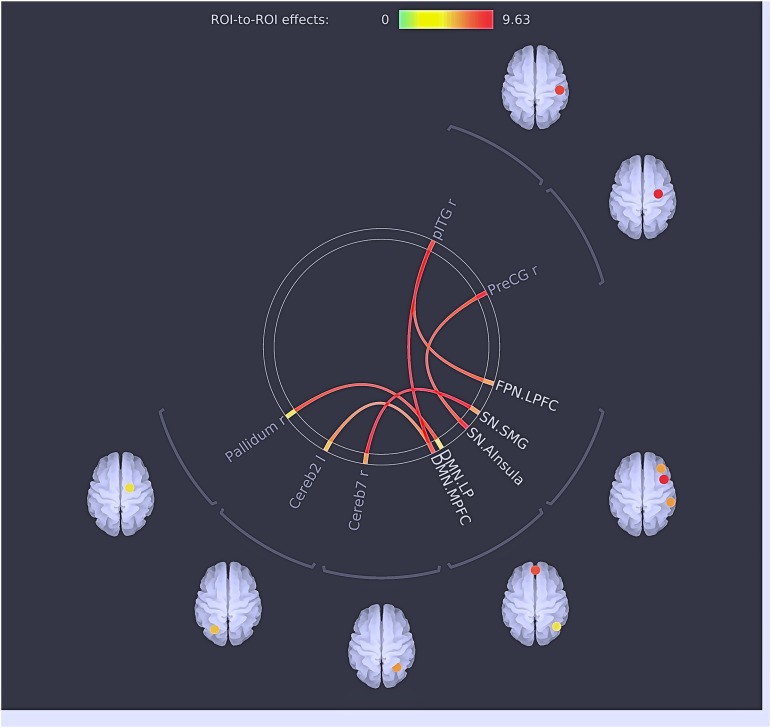
Results of multivariate analysis: the main effects of ROIs. The red lines represent positive connections/effects between ROI.

The main effect of the group was found between the seeds of the large-scale networks (LSNs) such as the default mode network (DMN); salience network (SN); fronto–parietal network (FPN); and the seeds in the cerebellum, thalamus, somato-motor, and temporal cortices (Figure 3 and Table 9).

**Table 9:** Results of the multivariate analysis (MANCOVA) with the cognitive cluster groups and healthy individuals and the scanner type as a covariate.

	Seed	ROI	*F*(3)(128)	*p*-uncorr	*p*-FDR
Main effect	DMN MPFC	pITG R	8.92	0.0000	0.0032
		Cereb 2L	6.28	0.0005	0.0408
	DMN LP	Pallidum R			
	FPN LPFC	pITG R	7.11	0.0002	0.0291
	SN SMG R	Cereb 7R	9.63	0.0000	0.0014
	SN AInsula L	PreCG	7.23	0.0002	0.0252

**FIGURE 4:**
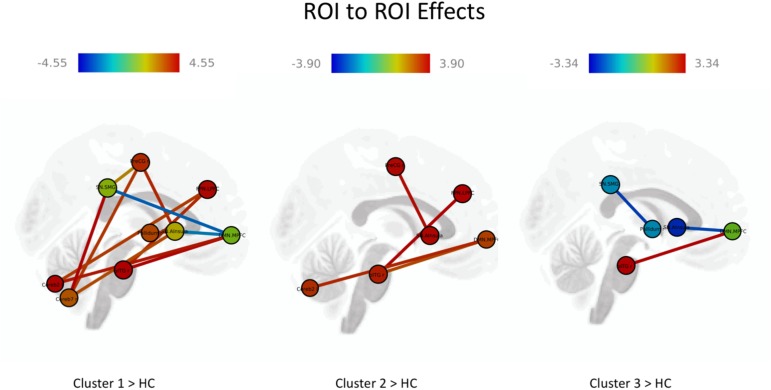
Results of the comparison between the cognitive cluster groups and healthy individuals. The red lines represent positive and blue line negative ROI-to-ROI significant effects. The darker red means a higher number of positive than negative effects and the darker blue a higher number of negative than positive effects.

*Post hoc* tests revealed significant between-group (clusters) differences, resulting in both hyper- and hypoconnectivity between the subcortical–cortical and cortical–cortical seeds (Figure 4 and Table 10). Clusters 1, 2, and 3 (patient groups) showed a similar pattern of strong positive connectivity between the MPFC and inferior temporal region compared to the HC. Cluster 1 and Cluster 2 differed from the HC in connectivity between the medial prefrontal cortex (MPFC, anterior DMN) and cerebellum; lateral prefrontal cortex (LPFC, FPN) and inferior temporal gyrus (IFG); and anterior insula cortex (part of the SN) and precentral gyrus (PreCG), being higher in the patient groups. In addition, as with Cluster 1, the differences between Cluster 3 and the HC were observed in the hypoconnectivity between the MPFC and anterior insula (FDR uncorr). Also, Cluster 1 demonstrated hyperconnectivity between the supramarginal gyrus (SMG, SN) and seeds in the precentral gyrus and cerebellum, as well as in the anterior insular part and the right pallidum and cerebellum compared to the HC. At the same time, hypoconnectivity between the MPFC and SMG and anterior insula (parts of the SN) was found. In contrast to the HC, Cluster 3 showed hypoconnectivity between the right SMG and the pallidum (FDR uncorr).

**Table 10:** *Post hoc* tests: between-group comparison of cluster groups vs. the group of healthy individuals.

	Seed	ROI	*T*(128)	*p*-uncorr	*p*-FDR
*C1 > HC*	SN SMG	Cereb 7R	4.55	0.0000	0.0001
		PreCG R	2.06	0.0416	0.0111
		DMN MPFC	-2.37	0.0193	0.0771
	SN Ansula R	PreCG R	3.42	0.0008	0.0067
		Pallidum R	3.15	0.0020	0.0081
		Cereb 7R	2.79	0.0062	0.0164
	FPN LPFC	pITG R	3.39	0.0009	0.0074
		Cereb 2L	3.06	0.0027	0.0108
	DMN MPFC	pITG R	4.32	0.0000	0.0003
		Cereb 2L	3.74	0.0003	0.0011
		SN AInsula R	-2.11	0.0367	0.0734
*C2v > HC*	DMN MPFC	Cereb 2L	2.92	0.0042	0.0335
	FPN LPFC	pITG R	3.90	0.0002	0.0012
	SN AInsula R	PreCG R	3.48	0.0007	0.0054
*C3 > HC*	DMN MPFC	pITG R	3.34	0.0011	0.0274
		SN AInsula R	-2.07	0.0400	0.1636
	SN SMG R	Pallidum R	-2.20	0.0299	0.2392

## Discussion

Based on the cluster analysis of their cognitive performance, we examined the cognitive profiles of FES subjects, and correlated these profiles with clinical variables and resting state brain connectivity measured with magnetic resonance images.

Our sample consisted of patients with first-episode early stage schizophrenia spectrum disorders, homogeneous in age, education, and gender. In contrast to other studies with chronic patients (Goldstein et al., 1998), we did not find any significant differences between clusters in external variables, such as demographic characteristics, DUP, dosage of medication, or subjective evaluation of their QOL. Moreover, we did not find differences in the sample between individuals with F20 and F23 diagnoses. We find these results consistent with more recent studies that also assessed a sample at first disease onset (e.g., Gilbert et al., 2014) or a sample of mixed psychotic disorders (Lewandowski et al., 2014).

The findings of this study confirmed in three predefined subgroups different cognitive patterns and levels of performance. The presence of various cognitive performance subgroups has been replicated in other schizophrenia studies, regardless of the methodological differences such as the composition of neuropsychological measures and study samples (Seaton et al., 1999; Gilbert et al., 2014). Subgroups of cognitive performance have also been found in first-degree relatives (Quee et al., 2014; Ohi et al., 2017; Rodriguez et al., 2018) and in other psychotic disorders (Lewandowski et al., 2014).

In our study all clusters scored at least 1SD below the average on Verbal Memory domain, and Cluster 2 scored above average in the attention domain. Despite the fact that the cluster analysis of the composition of affected cognitive domains within the clusters and the sample size in our study differed from other studies (e.g., Goldstein et al., 1998; Hill et al., 2002; Gilbert et al., 2014; Ohi et al., 2017), including our previous research (Rodriguez et al., 2017), we identified a subgroup with moderate deficit (Cluster 1), a mild (like-normal) cognitive subgroup (Cluster 2), and a subgroup with a generalized CD (Cluster 3) similarly to previously published findings (Seaton et al., 2001; Gilbert et al., 2014; Rodriguez et al., 2017). Gilbert et al. (2014) also found three clusters (in four domains) based on a defined cognitive decline under the 16th-1SD percentile in the cognitive domains. As in our work, they found a “near-normal functioning cluster,” but not a “normal cluster.” The mild (like-normal) group in our sample yielded a cognitive performance above the average in one domain (the attention domain in Cluster 2). There is a body of evidence suggesting that a highly functioning cognitive subgroup in schizophrenia may perform above average in at least one cognitive domain (Goldstein and Shemansky, 1995; Geisler et al., 2015). In the cluster with generalized and severe deficit (Cluster 3) one of the most impaired domains was verbal memory, and as expected in first episodes, this group was the least represented in the whole sample. The small representation of the sample in this cluster was expected, since the subjects are at the early stages of their illness, and are not affected by the collateral effects of the course of the illness (Volavka and Vevera, 2018). The longitudinal stability of cognitive cluster subtypes becomes later, with the trajectory of the disorder (Heinrichs et al., 1997).

Due the sample size, the number of domains (six), the variables to be analyzed, and in concordance with other previous studies (for e.g., Gilbert et al., 2014; Ohi et al., 2017; Rodriguez et al., 2017; Uren et al., 2017), we pre-defined only three clusters. An increase in the number of subjects analyzed could yield different cluster solutions in future research. Nevertheless, the number of cognitive clusters is more or less consistent across the different studies.

Cluster analyses based on cognitive performance determined the level of performance in our sample. In order to better reflect the external validity of the three subgroups, we analyzed them with neuroimaging variables. Several studies have attempted to do this, uncovering a consistent relationship within the cognitive profiles and brain structure (Heinrichs and Awad, 1993; Ohi et al., 2017), functional connectivity (He et al., 2013; Du et al., 2018), or both (Geisler et al., 2015). However, the majority of these studies did not select a homogenous sample.

In patients, the overall and substantial differences in whole brain connectivity in our study were found between LSNs such as the DMN, SN, FPN and cortical (somato-motor and temporal), subcortical structures (thalamus), and cerebellum. The dysconnectivity in LSNs and its relation to cognitive dysfunction in patients with schizophrenia was initially suggested by Bressler (2002) and later developed by Bressler and Menon (2010). Using multimodal structural and functional imaging approaches, they supported the view that cognition resulted from a dynamic interaction of distributed brain areas operating in LSNs. The patterns of dysconnectivity in three clusters encompass both the hyper- and hypoconnectvity of brain networks. Recent studies have frequently reported either hypoconnectivity or hyperconnectivity of brain networks in patients with schizophrenia and schizophrenia spectrum disorders (for a review see Dong et al., 2018). It seems that both decreased and increased connectivity result in the worse cognitive performance. As Skudlarski et al. (2010) suggest, increased connectivity may indicate an increased neural effort due to the presence of structural damage in certain brain structures, while a decrease in the connectivity would point to a different pattern or the decoupling of structural and functional connections (Skudlarski et al., 2010). In this regard, the increased connection between the medial frontal gyrus (DMN) and the IFG may be explained by potential structural changes in the temporal gyrus, a finding that is repeatedly described in schizophrenia patients (Onitsuka et al., 2004; Kuroki et al., 2007). At the same time, a hyperconnectivity pattern between the IFG and the medial frontal cortex (anterior seed of DMN) presumably indicates a lack of suppression (Seidman et al., 2014) exclusively pronounced in patients with CDs (Zhou et al., 2016). This assumption could be tested in a future study.

Cluster 1 and Cluster 2 shared a common hyperconnectivity between the MPFC (DMN) and the cerebellum. While examining the functional connectivity between the task-responsive parts of the cerebellum, Brissenden et al. (2016) concluded that cerebellum-to-cortex functional connectivity strongly predicted the pattern of cortical activation during attentional task performance. Increased connectivity between the DMN and cerebellar seeds found by Guo et al. (2015, 2018) in patients with schizophrenia and their siblings was suggested to be a potential endophenotype for schizophrenia. Another commonality that was shared by the relatively similar cognitive Clusters 1 and 2 is hyperconnectivity between the anterior insula cortex (SN) and the IFG. This particular connection was hypothesized by Tops and Boksem (2011) for the elaboration of attentional and WM processing. As they assume, the SN–IFG connection aims to facilitate fast and accurate responses, but it may cause slow responses, or it may interfere with the accuracy and speed of performance in the next trial when processing of the next stimulus follows prolonged elaborate processing. Altered connectivity between the anterior insula and auditory cortices has been shown to be significantly associated with cognitive impairment (Tian et al., 2018). Another pattern of increased connections between the anterior insula and the somatosensory cortex, and specifically the precentral gyrus, was reported by Tian et al. (2018). Although the authors did not draw parallels with symptoms or cognitive functioning, such a pattern may reflect talk-level control deficits and/or difficulties in focal attention processing (Nelson et al., 2010).

Frontal–parietal network connectivity, altered in both Cluster 1 and Cluster 2, is considered to be a flexible hub adapting to old and novel tasks (Fassbender et al., 2006; Zanto and Gazzaley, 2013) and is associated with cognitive control. Altered connectivity in schizophrenia patients was shown to be linked to WM deficits and notably to a failure of context-sensitive coupling (Nielsen et al., 2017). At the same time, fronto-temporal connections, as suggested by Kobayashi (2009), are associated with visual recognition and memory. Specifically, the ITG plays an important role in linking visual stimuli with a reward outcome.

In Clusters 1 and 3, we identified similar patterns of hypoconnectivity between the MPFC (DMN) and the anterior insula (SN). A similar finding was reported in the study of Manoliu et al. (2013) demonstrating a decreased temporal dependence of DMN activity on SN activity in patients with schizophrenia during acute psychosis. However, the study of Wang et al. (2015) reports the opposite results. With respect to cognitive performance, the degree of disruption of the SN that modulates the DMN and central executive networks correlates with lower cognitive performance in an aging population (Chand et al., 2017), although no evidence has been provided in schizophrenia.

Exclusively for Cluster 1, we identified hyperconnectivity between the FPN (LPFC) and the cerebellum. This is in line with the findings of Kim et al. (2017) who demonstrated an increased fronto-cerebellar connectivity (*r* = 0.57, *p* < 0.001). With respect to cognitive performance, together with the FPN, the cerebellum and specifically its posterior part play a role in high cognitive processing, i.e., motor control, subsequently updating actual and mental motor performance (Bonzano et al., 2016). Another finding that was prominent only in the first cluster is hypoconnectivity between the SMG and the DMN. The SMG supports the role in multiple cognitive domains such as visual word recognition in memory tasks (Stoeckel et al., 2009), behavioral switching (Jubault et al., 2007), or decision-making (Boorman and Rushworth, 2009). In schizophrenia, the SMG shows a significant gray matter reduction (Palaniyappan and Liddle, 2012), thereby affecting cognitive processing.

Hypoconnectivity of the SMN and basal ganglia (pallidum) was found exclusively in Cluster 3. Decreased activity of the pallidum has been reported to be associated with processing speed alterations in patients with schizophrenia (Mwansisya et al., 2013); however, in such an association it may impact VISM processing.

Taken together, using explorative correlation analysis of the whole brain connectome, we were able to identify three patterns of connectivity, specifically in the brain networks and seeds that are related to the interactions of the attentional and memory systems. These three clusters were associated with different constellations of CDs that differed mostly in attention and FLEX.

### Limitations

The main limitations in our study include the sample size for the cluster analysis and the unclear stability of the clusters over time. In a larger sample we could predefine more clusters, which could reveal better differences in cognitive performance. We analyzed a sample of patients with FES. Since the subjects are in their early stage, we do not know the course of their illness yet. The longitudinal stability of cognitive cluster subtypes becomes apparent later, with the trajectory of the disorder (Heinrichs et al., 1997). Our next study will attempt to cover these limitations. We will increase the sample size, and will assess the sample after 1 year in order to follow-up the course. Lastly, given that the clusters could not be directly matched to the whole brain connectivity patterns and were only investigated by comparing the groups in the form of an explorative analysis, the results should be interpreted with caution. Our limited sample size may have prevented us from finding smaller differences between the cluster groups, though by adding uncorrected results we highlighted the directionality (positive or negative) of the connectivity patterns in three cluster groups that impacted the cognitive performance (discussed above). Besides, the control group was matched only by age and gender, the education level was lower in patients. Indeed, the education level was shown to increase brain efficacy in healthy individuals (Marques et al., 2015). The effect of education will be considered in the future research.

In conclusion, the cluster solution confirms the prevalence of a cognitive decline in FESs with different patterns of cognitive performance, and different levels of severity. Moreover, separate behavioral cognitive subsets can be linked to patterns of brain functional connectivity.

## Data Availability

The datasets generated for this study are available on request to the corresponding author.

## Ethics Statement

The study was conducted at the National Institute of Mental Health, Czech Republic; the study protocol was approved by the Ethical Committee of the institution: Etická Komise NÚDZ. All of the study subjects signed an informed consent form before the enrolment.

## Author Contributions

MR designed the study and wrote the original protocol together with ZK, FS, and YZ. AD, JJ, PS, VV, and MH recruited the participants, and performed the neuropsychological and fMRI assessment. AC, BD, and YZ pre-processed the data and performed the statistical analysis. MR supervised the study. MR, YZ, and PM wrote the first draft of the manuscript and contributed to the data interpretation. All of the authors discussed the results and contributed to the final version of the manuscript and have approved it.

## Conflict of Interest Statement

The authors declare that the research was conducted in the absence of any commercial or financial relationships that could be construed as a potential conflict of interest.
